# Case Report: Efficacy of anlotinib and sintilimab in treating lung adenocarcinoma with RET fusion and PD-L1 expression

**DOI:** 10.3389/fphar.2024.1448291

**Published:** 2024-11-28

**Authors:** Hejing Bao, Jiani Zhang, Yuhuan Wang, Zhiting Chen, Xi Luo, Ting Li, Haoran Su, Hehong Bao, Xiaolong Cao, Liping Lin

**Affiliations:** ^1^ Department of Oncology, The Affiliated Panyu Center Hospital, Guangzhou Medical University, Guangzhou, Guangdong, China; ^2^ Cancer Institute of Panyu, Guangzhou, Guangdong, China; ^3^ Department of Oncology, Nanfang Hospital, Southern Medical University, Guangzhou, Guangdong, China; ^4^ Department of Psychosomatic Medicine, Chongqing University Three Gorges Hospital, Chongqing, China

**Keywords:** non-small cell lung cancer, RET fusion, high PD-L1 expression, anlotinib, sintilimab

## Abstract

We report a case of an advanced non-small cell lung cancer (NSCLC) patient with brain metastasis, RET fusion, and high expression of programmed death ligand 1 (PD-L1) at initial treatment. After receiving radiotherapy for the brain metastasis, the patient started with anlotinib and added immunotherapy with sintilimab. The patient had a good response to anlotinib and sintilimab treatment, tolerated the adverse reactions, and had a progression-free survival (PFS) of over 17 months. To the best of our knowledge, this is the first clinical case report in the literature describing the benefit of anlotinib and sintilimab treatment for non-small cell lung cancer with RET fusion and high PD-L1 expression. This study explores the biomarker selection for targeted therapy and combined immunotherapy in NSCLC patients.

## Highlights

• A patient with metastatic NSCLC with RET fusion and positive expression of PD-L1 responded well to combination therapy with anlotinib and sintilimab, with a PFS of over 17 months.
• Combination therapy with anlotinib and sintilimab for advanced RET fusion NSCLC patients may be a promising alternative to chemotherapy.
• This study explores the biomarker selection for anti-angiogenic targeted therapy and combined immunotherapy in NSCLC patients.

## Introduction

RET rearrangements occur in about 1%–2% of NSCLC patients, more commonly in adenocarcinoma patients (DoebeleRC et al., 2020; Ferrara et al., 2018). RET gene and other structural domains may undergo rearrangements (fusion), particularly with the kinesin family member 5B (KIF5B) and the coiled-coil domain containing 6 (CCDC6), resulting in overexpression of the RET protein (DoebeleRC et al., 2020; Ferrara et al., 2018; Gautschi et al., 2017). RET rearrangements occur in both smokers and non-smokers. RET rearrangements usually do not overlap with epidermal growth factor receptor (EGFR), ROS1, BRAF, MET ex14, and ALK gene mutations. However, some studies have shown that in rare cases, RET rearrangements may overlap with EGFR and KRAS mutations (Febbo et al., 2011; LeeSE et al., 2015). Data show that the response rate for single-agent immune checkpoint inhibitors (ICI) therapy in RET-positive metastatic NSCLC patients is approximately 6% (Mok et al., 2020).

Prasacitinib and selpercatinib are two highly selective RET inhibitors that have achieved a remarkable and sustained response rate of 60%–68% in RET-positive NSCLC patients (Gautschi et al., 2017; Febbo et al., 2011). In patients with metastatic RET fusion-positive NSCLC, selpercatinib treatment significantly prolongs the PFS compared to cisplatin-based chemotherapy alone or in combination with pembrolizumab. In a randomized phase 3 trial, the authors evaluated the efficacy and safety of selpercatinib as first-line therapy versus a control arm consisting of cisplatin-based chemotherapy alone or in combination with pembrolizumab. The median PFS was 24.8 months (95% CI, 16.9 to not estimable) in the selpercatinib group, compared to 11.2 months (95% CI, 8.8–16.8) in the control group (Hazard Ratio (HR), 0.46; 95% CI, 0.31 to 0.70; P< 0.001) (Zhou et al., 2023). However, the availability of these drugs in clinical settings is currently very limited, and they are mostly accessible through participation in clinical trials or through expanded access programs.

AL3818 (Anlotinib Hydrochloride) is a novel multi-targeted tyrosine kinase inhibitor (TKI) used for angiogenesis and proliferation signal transduction in tumors (Shen et al., 2018). Anlotinib’s main targets include receptor tyrosine kinases, vascular endothelial growth factor receptors 1–3, EGFR, fibroblast growth factor receptors 1–4, platelet-derived growth factor receptor α and β, and stem cell factor receptor. Additionally, it can inhibit tumor angiogenesis and tumor cell proliferation (Shen et al., 2018). In the II. phase ALTER 0302 study and III. phase ALTER 0303 study, the median PFS and overall survival (OS) of the anlotinib group were significantly longer than those of the placebo group (the median PFS was 4.8 months vs. 1.2 months, P < 0.0001; the median OS was 9.6 months vs. 6.3 months, P = 0.002) (Han et al., 2018a; Han et al., 2018b). There is currently no standard third-line treatment for advanced NSCLC in China, and the China Food and Drug Administration (CFDA) has approved anlotinib for the treatment of advanced NSCLC patients. Furthermore, a multi-center, single-arm, phase II study showed that anlotinib combined with paclitaxel and cisplatin as a first-line treatment for advanced esophageal squamous cell carcinoma (ESCC) has a manageable safety profile and sustained clinical responses (Li et al., 2022). A random phase IIB trial showed that anlotinib significantly prolonged the median PFS in patients with advanced soft tissue sarcoma (STS) (Chi et al., 2016). Anlotinib also showed good efficacy in patients with advanced thyroid medullary carcinoma and metastatic renal cell carcinoma (RCC) (Zhou et al., 2016; Brose et al., 2014).

Sintilimab is a fully human IgG4 monoclonal antibody against programmed death 1(PD-1) used in China (Zhang et al., 2022; Hoy, 2019; Zhang et al., 2020). It was first approved by the National Medical Products Administration (NMPA) of China for the treatment of patients with classic Hodgkin lymphoma who have relapsed or become refractory after at least two lines of systemic chemotherapy (Hoy, 2019; Zhang et al., 2020). Subsequently, sintilimab was approved by the NMPA for NSCLC combined with chemotherapy as a first-line treatment, and the results of phase 3 studies (ORIENT-11 and ORIENT-12) also provided support for the new approval of sintilimab as a first-line treatment regimen (National Medical Products Administration, 2021; Yang Y. et al., 2020; Zhou et al., 2021). Compared with chemotherapy alone, sintilimab combined with platinum-based chemotherapy has better anti-tumor efficacy and clinical benefits, and therefore has been approved in China and is undergoing a biologics license application in the United States. In addition, sintilimab has shown promising anti-tumor efficacy when combined with other regimens, such as docetaxel, cytokine-induced killer cell immunotherapy, radiation therapy, and anlotinib (Zhang et al., 2022).

Here, we report a case of a 68-year-old female with metastatic NSCLC who responded well to treatment with anlotinib and sintilimab, with RET fusion and positive PD-L1 expression, and to our knowledge, this is the first such clinical case report.

## Case presentation

**FIGURE 1:**
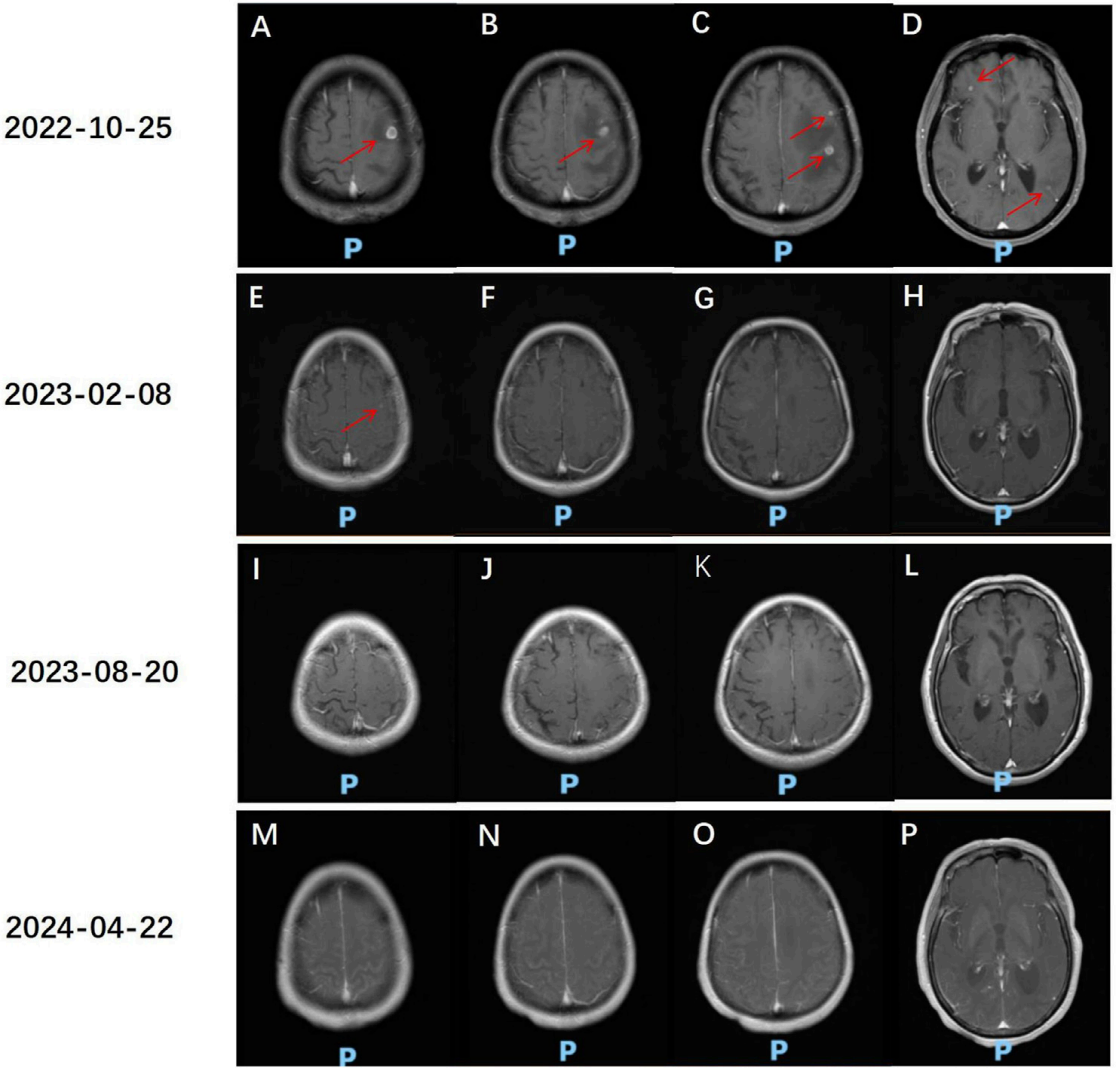
Head MR assessment of anlotinib and sintilimab treatment effects. **(A–D)** 25 October 2022, Head MR: Multiple small class circular abnormal signals are seen in the left parietal lobe of the occipital lobe and bilateral frontal lobes. The largest abnormal signal is located in the cortical layer of the left frontal lobe, with a size of approximately 9*10*9 mm (red arrow), and the boundary is clear. On T1WI, the abnormal signal is slightly lower in intensity, and on T2WI, it is slightly higher in intensity. On the enhanced scan, the lesion shows nodular and ring-like enhancement. There is a band of long T1 and T2 signal change in the surrounding brain parenchyma, which is more obvious on the left frontal lobe. **(E–H)** Head MR comparison with the previous film on 25 October 2022: The multiple small class circular abnormal signals in the left parietal lobe and bilateral frontal lobes have decreased and shrunk compared to the previous examination. A few nodular abnormal signals are seen in the superior parieto-temporal lobe on T1WI, with slightly higher intensity on T2WI. The boundary is clear. On the enhanced scan, the lesion shows nodular enhancement, with a larger diameter of approximately 4 mm (red arrow). **(I–L)** Head MR comparison with the previous film on 20 August 2023: The multiple small class circular abnormal signals in the left parietal lobe and bilateral frontal lobes have decreased and shrunk compared to the previous examination. The nodular abnormal signal in the superior parieto-temporal lobe is not visible. **(M–P)** Head MR comparison with the previous film on 22 April 2024: The multiple small class circular abnormal signals in the left parietal lobe and bilateral frontal lobes are not visible.

**FIGURE 2:**
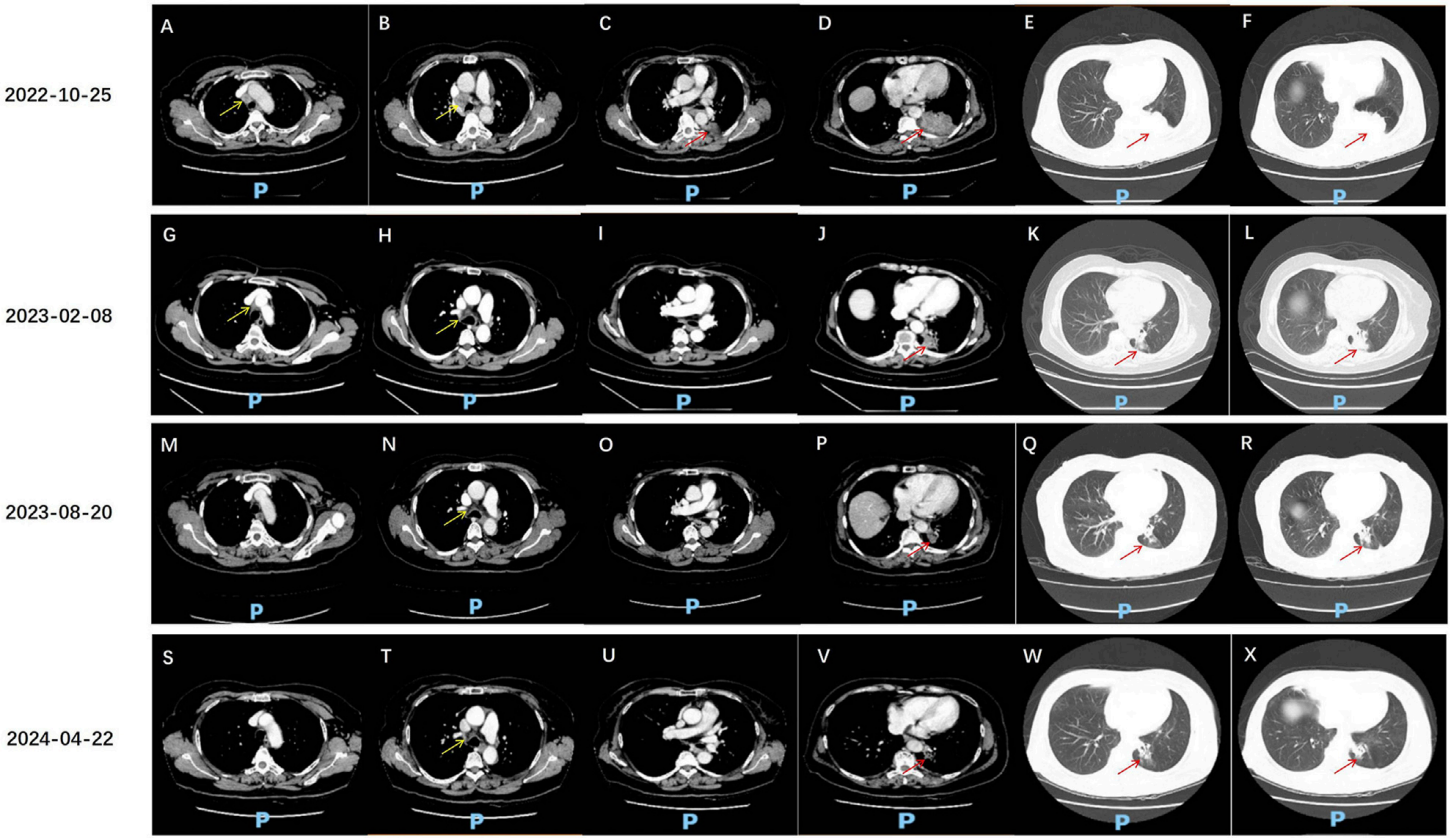
Chest CT assessment of anlotinib and sintilimab treatment effects. **(A–F)** 25 October 2022, chest CT: A solid density mass lesion is visible in the lower lobe of the left lung, with a clear boundary, and the cross-sectional size is approximately 59*45 mm (red arrow). The edge of the mass is lobulated and tightly adherent to the pleural membrane of the rib cage. The mass obstructs part of the bronchus. Enhanced scanning shows uneven enhancement. Multiple enlarged lymph nodes are seen in the left lung hilum and mediastinum, with the largest located in the 4R group of the mediastinum, measuring approximately 27*20 mm (yellow arrow), with visible enhancement. Some larger lymph nodes show areas of no enhancement due to necrosis. **(G–L)** 8 February 2023, chest CT: A solid density mass lesion is visible in the lower lobe of the left lung, which is smaller than before, with the current cross-sectional size of approximately 39*25 mm (red arrow, previously 59*45 mm). Multiple enlarged lymph nodes are seen in the left lung hilum and mediastinum, with the largest located in the 4R group of the mediastinum, measuring approximately 15*10 mm (yellow arrow, previously 27*20 mm). Uneven enhancement is seen. **(M–R)** 20 August 2023, chest CT: A solid density mass lesion is visible in the lower lobe of the left lung, which is smaller than before, with the current size of approximately 27*22 mm (red arrow, previously 39*25 mm). Multiple enlarged lymph nodes are seen in the left lung hilum and mediastinum, with the largest measuring approximately 15*10 mm (yellow arrow, similar to before). **(S–X)** 22 April 2024, chest CT: A solid density mass lesion is visible in the lower lobe of the left lung, with a clear boundary, and the size is smaller than before, measuring approximately 19*18 mm (red arrow, previously 27*22 mm). Multiple enlarged lymph nodes are seen in the left lung hilum and mediastinum, with the largest measuring approximately 15*10 mm.

**FIGURE 3:**
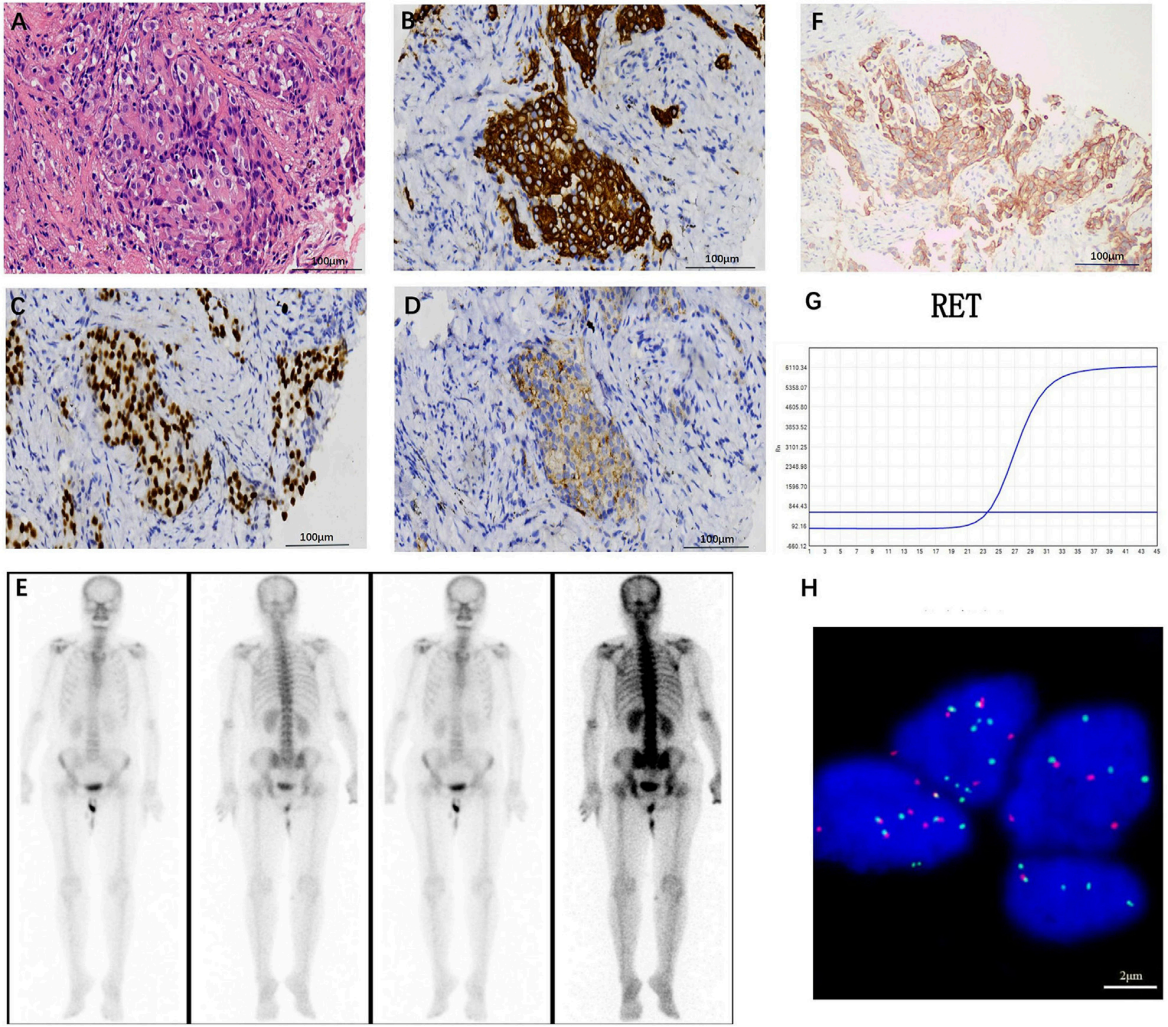
**(A)** Hematoxylin-eosin staining (HE) staining image (original magnification, ×200), left lung invasive adenocarcinoma, biopsy tissue predominantly solid in appearance, with tumor cells growing in small clusters, large and deeply stained nuclei, and mitotic figures visible. Immunohistochemical results suggest positive expression of CK7 **(B)**, TTF-1 **(C)**, and NapsinA **(D)** (original magnification, ×200). **(E)** Whole-body bone scan shows no significant abnormalities. **(F)** PD-L1 expression in tumor cells is as high as TPS 80% (original magnification, ×200). **(G)** RT-PCR identification indicates the presence of a KIF5B-RET gene fusion. **(H)** Separate fluorescence *in situ* hybridization (FISH) analysis indicates RET fusion (original magnification, ×1,000), with 54 positive cells and 46 negative cells, a positive cell ratio of 54%, and positive cells being cells with red-green signal separation or solitary red signal.

The patient, a 68-year-old female, underwent a computed tomography (CT) scan at one hospital in June 2022, which suggested a lung lesion, but she did not seek medical attention. The patient’s Eastern Cooperative Oncology Group (ECOG) performance status was 1. On 25 October 2022, she presented with a left-sided mouth droop and slurred speech, accompanied by weakness in her right limb. Head Magnetic Resonance Imaging (MRI) with contrast enhancement suggested multiple metastatic brain tumors in the left occipital lobe and bilateral frontal lobes (Figures 1A–D). The largest tumor was located in the left frontal lobe, measuring approximately 9*10*9 mm, with the left frontal lobe showing more extensive involvement (Figures 1A–D). Chest CT with contrast enhancement showed a lesion in the left lower lobe, suggestive of lung cancer, with a cross-sectional size of approximately 59*45 mm, with a shallow lobulated margin, and multiple lymph nodes in the left hilum and mediastinum, suggestive of metastasis, the largest located in the 4R group of the mediastinum, measuring approximately 27*20 mm (Figures 2A–F). On 2 November 2022, a biopsy was performed on the left lung lesion under ultrasound guidance in real-time imaging, with two specimens obtained for examination. Histopathological examination revealed (left lung lesion) invasive adenocarcinoma, with the biopsy specimen predominantly solid (Figure 3A). Immunohistochemical results showed CK7 (+), TTF-1 (+), NapsinA (+), P40 (−), CK5/6 (−) (Figures 3B–D). A whole-body bone scan did not show any significant abnormalities (Figure 3E). The final diagnosis was left lung adenocarcinoma with metastasis to the lung hilum, mediastinal lymph nodes, and brain, with TNM staging of T3N3M1b IVA (AJCC 8th edition).

Through cloning the Dako 22C3 pharmDx antibody test, it was found that the PD-L1 was expressed at a tumor proportion score (TPS) rate of up to 80% in tumor cells (Figure 3F). With the consent of the patient and his family, the Yukang 488 tumor-related gene was detected using tumor tissue and matched blood samples (OrigiMed, a College of American Pathologists–accredited laboratory, Yukang medical laboratory, shenzhen, China) and analyzed for genomic alterations, including single nucleotide variants, short insertions and deletions, copy number variants (CNVs), and gene rearrangements. Second-generation sequencing showed that the patient had four somatic mutations: a RET KIF5B exon15-RET exon12 fusion (K15:R12, abundance: 2.7%), which was further confirmed by Lung Cancer PCR Multigene Companion Diagnostic Kit (Maifittier ™) (Maijing Gene Medical Technology Co., LTD., Guangzhou, China) (Figure 3G) and ZytoVision *in situ* hybridization assay (Figure 3H) and telomerase reverse transcriptase (TERT), succinate dehydrogenase A (SDHA) and TRIO gene amplification. The TMB was 1.2 Muts/Mb (Table 1).

**TABLE 1:** NGS results of the NSCLC patient with RET fusion and High expression of PD-L1.

NGS	Results	Abundances
RET	KIF5B exon15-RET exon12 fusion	—
TERT	Amplification	25.4 CNV
SDHA	Amplification	9 CNV
TRIO	Amplification	21 CNV
TMB	1.2 muts/Mb	
MSI	MSS	

After radiotherapy was administered to the brain metastatic lesion, the radiation dose for the brain metastatic tumor was DT: PGTV42Gy/7F. The monthly cost of RET targeted therapy was approximately $9,000, while the monthly cost of traditional chemotherapy was about $700. Due to being unable to afford the high cost of RET targeted therapy, the patient refused it, and also due to concerns about the toxic effects of chemotherapy, the patient refused oral or intravenous chemotherapy. After referencing the NCT03628521 study published in JTO in 2021 (Chu et al., 2021), the patient was treated with anlotinib 8 mg po qod combined with sintilimab 200 mg d1 q3w for 17 cycles from 10 November 2022, to 22 April 2024. On 8 February 2023, the disease response was considered a partial response (PR) according to Response Evaluation Criteria in Solid Tumors (RECIST) version 1.1, with the findings showing that the multiple brain metastases in the left occipital lobe and bilateral frontal lobes had decreased and shrunk compared to the previous examination. Currently, there is a small metastasis in the left parieto-temporal lobe (Figures 1E–H). The lesion in the lower lobe of the left lung, which was treated, has shrunk compared to the previous examination, with the cross-sectional size currently being approximately 39*25 mm (compared to approximately 59*45 mm previously), and multiple enlarged lymph nodes in the left lung hilum and mediastinum, with sizes approximately 15*10 mm (compared to approximately 27*20 mm previously) (Figures 2G–L). On 20 August 2023, the disease response was considered PR (Figures 1I–L, 2M–R). On 22 April 2024, the disease response was considered PR (Figures 1M–P, 2S–X). The patient’s symptoms of limb weakness improved significantly after treatment and they were able to engage in normal activities. On 27 September 2023, the patient underwent a thyroid function test, with the thyroid-stimulating hormone (TSH) level being 25.635 μmol/L, indicating hypothyroidism, which was treated with thyroid hormone replacement therapy. Other adverse reactions included grade 2 hypertension and hoarseness of voice, which improved after antihypertensive treatment. The entire treatment process of the patient is shown in Supplementary Figure S1.

## Discussion

Although chemotherapy is the foundation of treatment for advanced lung cancer, many patients cannot tolerate this treatment with significant toxicity. This part of patients may not worry about the toxicity of chemotherapy if they have sensitive mutations, but the high cost of targeted therapy for rare mutations brings about significant “economic toxicity” (Yi et al., 2024). In September 2022, sepitinib was first approved in China, but it was not included in the national health insurance and could not be reimbursed, and its high cost made many Chinese patients hesitant. While, the cost of anlotinib combined with sintilimab was similar to that of traditional chemotherapy, at around $700 per month. What choices do these NSCLC patients with rare mutations have? We report a case of a patient with RET fusion and high PD-L1 expression who achieved satisfactory efficacy with immune-combined anti-angiogenic targeted therapy. This may provide a new option for these patients.

Current reports show that most RET fusion patients seem to be insensitive to immunotherapy. Sarfaty et al. (2017); Baglivo et al. (2020); Knetki-Wróblewska et al. (2022) conducted a series of studies on RET rearrangement lung adenocarcinoma (LADC) patients. The tumors in the patients showed positive staining for PD-L1, but were not responsive to pembrolizumab (Sarfaty et al., 2017; Baglivo et al., 2020; Knetki-Wróblewska et al., 2022). Offin et al. (2019) conducted a study involving 13 patients with advanced RET rearrangement NSCLC. All patients had a median PFS of 3.4 months (95% CI, 2.1–5.6 months). However, the PFS duration was shorter for patients with higher PD-L1 expression levels (50% and 30%), which were 1.3 months and 2.5 months, respectively. Guisier et al. (2020) identified 9 RET translocation patients in a real-world setting. Before receiving ICI, the patients had only received first-line treatment. The median PFS was 7.6 months, the median duration of response (DOR) was 4.7 months, and the objective response rate (ORR) was 38%. Bhandari et al. (2021) reported that 69 patients with RET fusion underwent immunotherapy as either first-line or second-line treatment. The median PFS for patients receiving immunotherapy as first-line treatment was 4.2 months (95%CI 1.4–8.4), and the mOS was 19.1 months. For the 12 patients who received chemoimmunotherapy as first-line treatment, the mPFS was 5.4 months, and the mOS was 19.1 months (6.9-NR); the ORR was 70%. Meng et al. (2022) reported that 49 patients with RET fusion-positive NSCLC received 92 treatments, with 26 (28.3%) based on ICI. In the ICI-based treatment group, PD-L1 expression level did not affect the mPFS of ICI [PD-L1 (+) vs. PD-L1(−): 4.7 (95%CI 1.8–9.0) months vs. 7.6 (95%CI 1.1–14.0) months, p = 0.910].

There have also been some reports of single-agent ICI sensitivity. Baby et al. (2021) recorded a case of metastatic RET rearrangement NSCLC with 100% positive PD-L1 expression, in which the patient achieved a sustained CR of 29 months after one-line pembrolizumab treatment for 8 months. Riudavets et al. (2021) reported a case of RET rearrangement NSCLC with PD-L1 expression of 90%, who achieved complete remission with single-agent ICI in the second-line treatment. The patient initially responded to pemetrexed and cisplatin, but later progressed. Then, pembrolizumab was started, and a sustained response lasting over 9 months was reported at the time of reporting. Nakasuka et al. (2021) reported the positive impact of pembrolizumab on 90% of PD-L1 positive advanced NSCLC patients with the CCDC6-RET fusion gene and NF1/TP53 mutations, but the patient discontinued ICI treatment after 8 months of stable disease due to the discovery of laryngeal cancer. There have also been reports of ICI being effective for locally advanced NSCLC neoadjuvant treatment, Dai et al. (2024) reported a case of a IIIA-stage pulmonary adenocarcinoma patient with a RET fusion gene and high PD-L1 expression who achieved pathological complete response after receiving neoadjuvant chemoimmunotherapy. The patient has survived for 12 months and has not had any recurrence or metastasis postoperatively. There have also been reports of ICI being effective for RET fusion in other cancer types, Yang X. et al. (2020) reported a case of a metastatic hepatocellular carcinoma (HCC) patient with RET amplification, high tumor mutation burden, and positive for PD-L1 (5%). The patient had additional mutations including TP53 c.673delC and TERT p.C228T. The patient had a good response to cabozantinib and nivolumab combination therapy, with a PFS of over 25 months.

Although the patient had high PD-L1 expression, the efficacy of immune monotherapy in advanced NSCLC patients with combined RET fusion is not certain. Under the premise that the patient has refused intravenous chemotherapy due to being unable to afford targeted therapy, we recommend that the patient receive sintilimab combined with anlotinib treatment. According to the phase 1b trial NCT03628521 study reported that the combination of sintilimab and anlotinib as a first-line treatment showed good efficacy, durability, and tolerability in advanced NSCLC patients who had not developed sensitive mutations (Chu et al., 2021). Compared with the use of anlotinib alone (9.2%) or sintilimab alone (20%), the ORR of patients receiving this combination treatment was superior (72.7%) (Chu et al., 2021). This response was comparable to the efficacy of sintilimab combined with chemotherapy in first-line advanced or metastatic NSCLC (non-squamous and squamous cell carcinoma were 68.4% and 64.7%, respectively) (Chu et al., 2021). Therefore, sintilimab combined with anlotinib may be a promising chemotherapy-free option for initial treatment of advanced NSCLC patients. Sintilimab combined with anlotinib has shown good therapeutic effects and tolerable toxicity in extensive-stage small cell lung cancer, cervical cancer, endometrial cancer, liver cancer, and bile duct cancer (Ma et al., 2024; Xu et al., 2022; Wei et al., 2022; Chen et al., 2022; Jin et al., 2023).

The reason why immune checkpoint inhibitors are effective when combined with anti-angiogenesis drugs in anti-tumor therapy is that immune checkpoint inhibitors can transform the immune microenvironment that favors tumor growth into one that inhibits tumor growth, while anti-angiogenesis drugs can cut off the nutrient supply for tumor growth, which cannot be separated from the VEGF secreted by the tumor itself. VEGF, in addition to promoting tumor angiogenesis, can also promote rapid tumor growth and inhibit the function of immune cells (T cells, NK cells, DC cells, etc.) (Song et al., 2020; Lee et al., 2020). Therefore, the combination of immunotherapy and anti-angiogenesis therapy has a synergistic effect in treatment, including controlling tumor angiogenesis, normalizing blood vessels, allowing more PD-1 monoclonal antibodies to enter the tumor microenvironment and exert their effects (Huinen et al., 2021); inhibiting VEGF at the same time can also upregulate PD-L1 expression, making immunotherapeutic drugs more effective; and blocking VEGF also releases immune cells from inhibition, enhancing the body’s immune function. In the first-line treatment of advanced tumors, pembrolizumab combined with lenvatinib (Gutierrez et al., 2023; Llovet et al., 2023) and atezolizumab combined with bevacizumab (Finn et al., 2020) have also achieved very good results, even better than those achieved with chemotherapy combined.

The patient’s next-generation sequencing (NGS) results showed a RET fusion, accompanied by amplifications of TERT, SDHA, and TRIO. RET fusion in advanced NSCLC was not discovered until 2012, and in early trials, multi-kinase inhibitors (MKIs) were primarily selected, which are other RTK and/or kinase inhibitors that inhibit RET-RTK drugs (such as VEGFR, BRAF, ALK, and EGFR, etc.) (Takahashi et al., 1985; Rocco et al., 2023). There are no reports of anlotinib treatment in advanced NSCLC RET fusion patients. TERT is the main component of the telomerase complex, and TERT amplification is often found in early lung cancer (Gaspar et al., 2018). Liu et al. showed that the prognostic significance of TERT CNV, indicating that TERT is related to a 35% reduction in the risk of LUAD progression (Liu et al., 2015). TERT gene activation and telomerase activation are related to cell proliferation and regulation (Mc Leer et al., 2022), and therefore, anti-cancer drugs that target TERT amplification can be used to treat cancer (Yang et al., 2021). However, the FDA has not yet approved anti-cancer drugs targeting the TERT gene. SDH is a mitochondrial enzyme that obtains electrons and transfers them through four subunits (SDHA, SDHB, SDHC, and SDHD) (Moreno et al., 2020). SDHA mutations are related to the onset of neurodegenerative diseases and are associated with the etiology of paraganglioma (PGL) and gastrointestinal stromal tumor (GIST) (Parfait et al., 2000; Nannini et al., 2020). TRIO is a Rho guanine nucleotide exchange factor (RhoGEF) that is critical to glutamatergic synaptic function and is associated with autism spectrum disorder/intellectual disability (ASD/ID) (Tian et al., 2021). There are no definitive reports on the relationship between SDHA and TRIO and lung cancer at present. The patient did not show resistance to ICI therapy, which may also be related to coexisting TERT amplification. The combined ICI and MKIs treatment produces effective antitumor effects by inhibiting tumor proliferation and regulation (Watkins et al., 2020), which needs further confirmation by subsequent studies.

In this case, the combination of anlotinib and sintilimab was used for first-line treatment, and the immune- and TKI-mediated toxicity was tolerable during treatment. The NCT03628521 study reported a treatment-related adverse event rate of 54.5%, with grade 3 or higher events predominating (22 out of 52 patients, 9.1%) (Chu et al., 2021). No grade 4 treatment-related adverse events were observed, and one case of grade 5 immune-related pneumonitis was reported (Chu et al., 2021). In a retrospective, multicenter study, ICI was used as second-line or later treatment for 9 patients with advanced RET fusion NSCLC, and the researchers reported that 10% of the enrolled patients experienced 3–5 grade immune-mediated adverse events (AEs) (most commonly colitis) (Meng et al., 2022). In our case, the patient mainly experienced grade 2 hypertension and grade 1 thyroid dysfunction, which were manageable with thyroid hormone replacement therapy and antihypertensive treatment. No other immune-related adverse reactions such as immune-related colitis were observed, and the toxicity was tolerable. However, there were no cases of using RET-TKI in this report, and the related toxicity risk associated with concurrent or sequential treatment with ICI and RET-TKIs is very limited, and further exploration is needed.

## Conclusion

In summary, we provide the first direct evidence that RET fusion and PD-L1 highly positive advanced NSCLC responds well to the combination of anlotinib and sintilimab, with a progression-free survival exceeding 17 months. Anlotinib and sintilimab combination therapy for advanced RET fusion NSCLC patients may be a promising chemotherapy-free option. However, the proportion of patients with high expression of PD-L1 in RET fusion patients is only 17%–18% (Zhou et al., 2023). Future studies with larger cohorts are needed to further confirm the efficacy of treatment regimens and elucidate the potential clinical feasibility of immunotherapy combined with MKIs in these patients.

## Summary

This case report reports for the first time the benefits of anlotinib and sintilimab for RET fusion and PD-L1 positive non-small cell lung cancer patients.

## Data Availability

The original contributions presented in the study are included in the article/Supplementary Material, further inquiries can be directed to the corresponding authors.

## Supplementary material

SUPPLEMENTARY FIGURE 1 Timeline of the treatment process.

## Glossary

NSCLC: Non-small cell lung cancer
PD-L1: Programmed death ligand 1
PFS: Progression-free survival
KIF5B: Kinesin family member 5B
CCDC6: Coiled-coil domain containing 6
ICI: Immune checkpoint inhibitors
HR: Hazard Ratio
TKI: Tyrosine kinase inhibitor
EGFR: Epidermal growth factor receptor
OS: Overall survival
CFDA: China Food and Drug Administration
ESCC: Esophageal squamous cell carcinoma
STS: Soft tissue sarcoma
RCC: Renal cell carcinoma
PD-1: Programmed death 1
NMPA: National Medical Products Administration
CT: Computed tomography
ECOG: Eastern Cooperative Oncology Group
MRI: Magnetic Resonance Imaging
TPS: Tumor proportion score
CNVs: Copy number variants
PR: Partial response
RECIST: Response Evaluation Criteria in Solid Tumors
TSH: Tthyroid-stimulating hormone
LADC: Lung adenocarcinoma
DOR: Duration of response
ORR: Objective response rate
HCC: Hepatocellular carcinoma
NGS: Next-generation sequencing
MKIs: Multi-kinase inhibitors
TERT: Telomerase reverse transcriptase
SDHA: Succinate dehydrogenase A
RhoGEF: Rho guanine nucleotide exchange factor
ASD/ID: Autism spectrum disorder/intellectual disability
PGL: Paraganglioma
GIST: Gastrointestinal stromal tumor
FISH: Fluorescence *in situ* hybridization
HE: Hematoxylin-eosin staining
AEs: Adverse events.
