# Supplementary figures and images for: Case Report: Efficacy of anlotinib and sintilimab in treating lung adenocarcinoma with RET fusion and PD-L1 expression
Source: Front Pharmacol. 2024 Nov 28;15:1448291. doi: 10.3389/fphar.2024.1448291 (PMC11635301; doi:10.3389/fphar.2024.1448291)

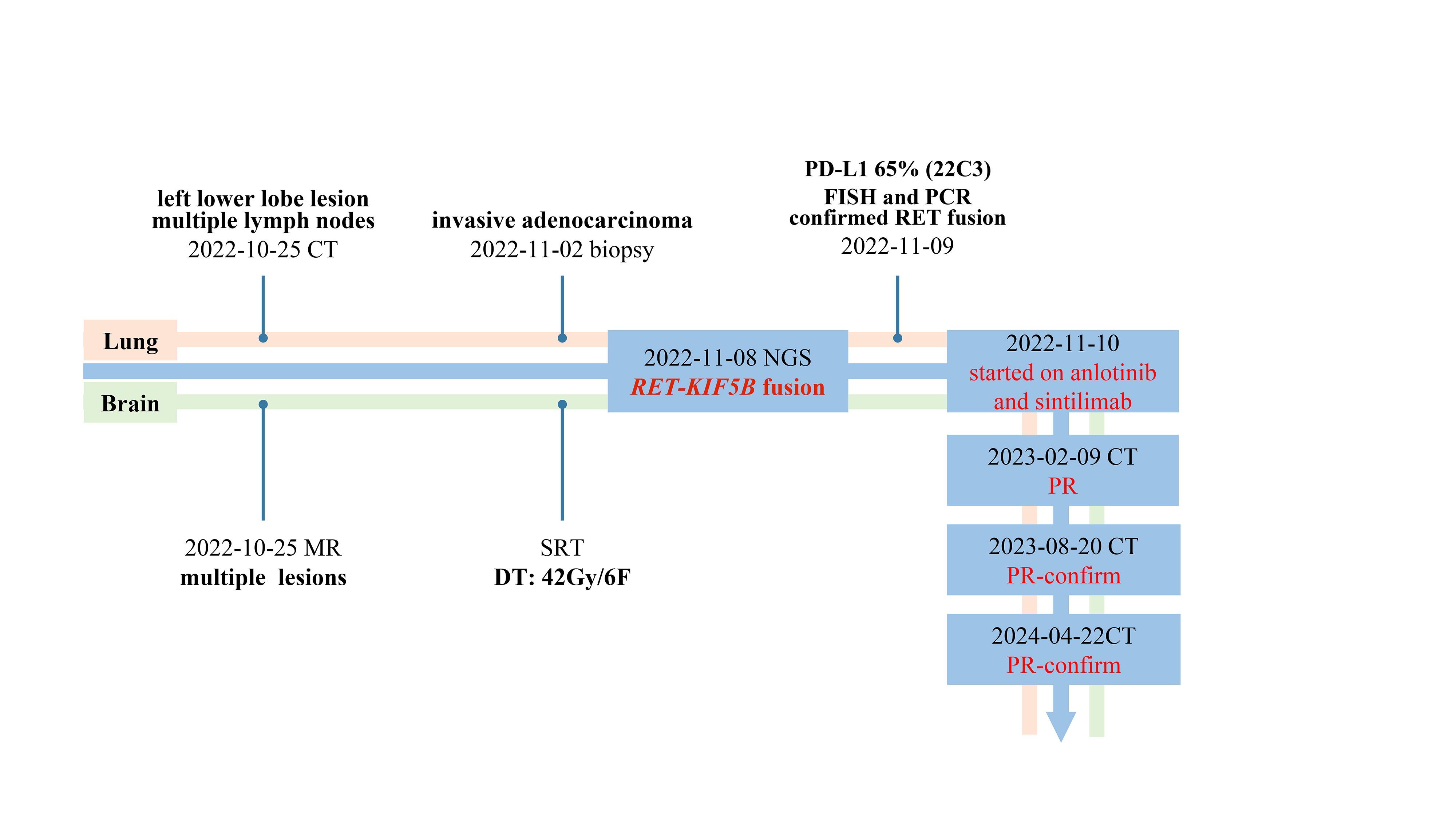

Supplement: Supplementary file 2 [file Image1.JPEG]
